# Morphological and functional parameters of left atrial appendage play a greater role in atrial fibrillation relapse after radiofrequency ablation

**DOI:** 10.1038/s41598-020-65056-3

**Published:** 2020-05-15

**Authors:** Xin Tian, Xue-Jing Zhang, Ying-Fang Yuan, Cai-Ying Li, Li-Xia Zhou, Bu-Lang Gao

**Affiliations:** 1 Department of Medical Imaging, The Second Hospital of Hebei Medical University, Shijiazhuang, China; 2 Department of Medical Research, Shijiazhuang First Hospital, Hebei Medical University, Shijiazhuang, China

**Keywords:** Cardiac device therapy, Radiography

## Abstract

This study was to quantitatively investigate the role of morphological and functional parameters of the left atrium (LA) and left atrial appendage (LAA) with 256-slice spiral computed tomography (CT) in the recurrence of atrial fibrillation (AF) after radiofrequency ablation (RFA). Eighty-three patients with AF who underwent RFA for the first time were divided into the recurrence (n = 27) and non-recurrence (n = 56) groups. All patients underwent a 256-slice spiral CT examination before the operation. The clinical data and quantitative measurement of the morphology and functional parameters of the LA and LAA were analyzed, including the maximal and minimal volume, ejection fraction and volume, and volume strain of LAA and LA (LAAVmax, LAAVmin, LAAEF, LAAEV, and LAA-VS, LAVmax, LAVmin, LAEF, LAEV and LA-VS, respectively). The CHA2DS2-VASc score and the proportion of patients with heart failure were significantly (P < 0.05) higher in the recurrence than non-recurrence group. The LAAVmax, LAAVmin, LAVmax, LAVmin, LAAV and LAV were all significantly greater in the recurrence than non-recurrence group (P < 0.05), and the perimeter, major and minor axes of LAA orifice and LAA depth were also significantly greater in the recurrence than non-recurrence group. The LAAEF, LAEF and LAA-VS were significantly (P < 0.05) lower in the recurrence than non-recurrence group (P < 0.05). Heart failure, CHA2DS2-VASC score, LAEF, LAV, LAAEF and LAA-VS were univariately significant (P < 0.05) risk factors for AF recurrence after ablation. Multivariate analysis revealed LAAEF (HR: 0.790, 95% CI: 0.657–0.950, P = 0.012) and LAAV (HR: 1.160, 95% CI: 1.095–1.229, P <0.001) to be two significant independent predictors of recurrence. ROC curve analysis showed that LAAEF <44.68% had the highest predictive value for recurrence after radiofrequency ablation, with the sensitivity of 90% and specificity of 67.4%, whereas LAA volume >9.25 ml had the highest predictive value for AF recurrence after RFA, with the sensitivity of 85.2% and specificity of 67.9%. In conclusion, the volume of left atrium, volume and morphology of left atrial appendage have all significantly increased while the ejection fraction and volume strain of left atrium and left atrial appendage have both significantly decreased in recurrence than in non-recurrence after radiofrequency ablation. The ejection fraction and volume of left atrial appendage are significant independent predictors of atrial fibrillation recurrence after radiofrequency ablation.

## Introduction

Atrial fibrillation (AF) is the most common cardiac arrhythmia and affects 1%–2% of the general population, with the incidence increased with age^1^. AF causes abnormal left atrial hemodynamics, subsequent thrombosis, myocardial infarction and heart failure, resulting in increased risks of stroke and mortality. Radiofrequency ablation aiming for pulmonary vein isolation has become an established option for drug-refractory AF, but with high rates of recurrence following ablation (accounting for 10%–30%)^2^, with the success rate of pulmonary vein isolation varying between 50% and 80%^3^. It has been shown that left atrial appendage (LAA) is one of the focal potential sources to spontaneously trigger AF, with the left atrium being another possible source of recurrence^4^. Left atrial volume enlargement and hypofunction are risk predictors after radiofrequency ablation of AF because AF causes remodeling and fibrosis of the left atrium. Dysfunction is a more sensitive recurrence predictor than size. Patients with a normal size of left atrium but reduced left atrium ejection fraction (LAEF) may experience recurrence after ablation^5–7^. Meanwhile, LAA function is also important in predicting recurrence but is largely neglected. The roles of the left atrium and LAA in AF recurrence have not been clearly stated and may be controversial. Recently, some new methods and markers for left atrium and LAA dysfunction have been investigated including assessment of myocardial strain, which is a relatively novel and promising cardiac imaging technique. Strain has been proven to be a risk factor stratification method for various cardiac disorders such as heart failure and coronary artery disease. It has been measured with ultrasound and magnetic resonance imaging, but no studies have been performed using computed tomography (CT) imaging to evaluate strain. We hypothesized that the volumetric and functional parameters of left atrium and LAA including the volume strain of left atrium and LAA evaluated with CT scan could be used as effective predictors for recurrence of AF after radiofrequency ablation. This study was consequently performed using a 256-slice spiral CT scanner to measure the volumetric and functional parameters of left atrium and LAA for predicting possible recurrence of AF following radiofrequency ablation.

## Materials and methods

This study was approved by the ethics committee of the Second Hospital, Hebei Medical University with all patients given signed informed consent. All methods were performed in accordance with the relevant guidelines and regulations. Patients who had undergone catheter radiofrequency ablation for AF for the first time in our hospital between October 2016 and June 2017 were enrolled. The inclusion criteria were patients who had radiofrequency ablation for AF which was clinically confirmed and diagnosed by physical examination and electrocardiogram, with preoperative 256-slice spiral CT examination but no serious procedural complications. The exclusion criteria were poor quality of CT angiography (CTA) images for measuring the volume of the left atrium and LAA, contraindication for CTA examination, contrast agent allergy, pacemaker implantation, valvular and congenital heart disease. The basic data, complications, types and duration of AF were collected. The risk of thromboembolism in patients with AF was assessed with the CHA2DS2-VASc score^8^.

### Examination methods

Philips 256-slice spiral CT scanning was performed in patients with AF. Before scanning, patients were trained in breath holding, and metoprolol was used to decrease the heart rate. Non-ionic contrast agent iohexol (350 mg I/ml, 0.8 ml/kg) was injected intravenously with a two-barrel high-pressure syringe. The scanning range was from 0.5 cm below the tracheal bifurcation to the diaphragm of the heart. Retrospective electrocardiogram (ECG) gating technique was used. The scanning parameters were: tube voltage 80–120 kV, tube current 280–350 mAs/rp, collimation 128*0.625, pitch 0.18, matrix 512*512, rotation time 330 ms, and scanning field 250 mm. The scanning voltage and current were generally adjusted according to the body mass index (BMI) of the patient to reduce scanning dose.

### Imaging postprocessing

**Figure 1:**
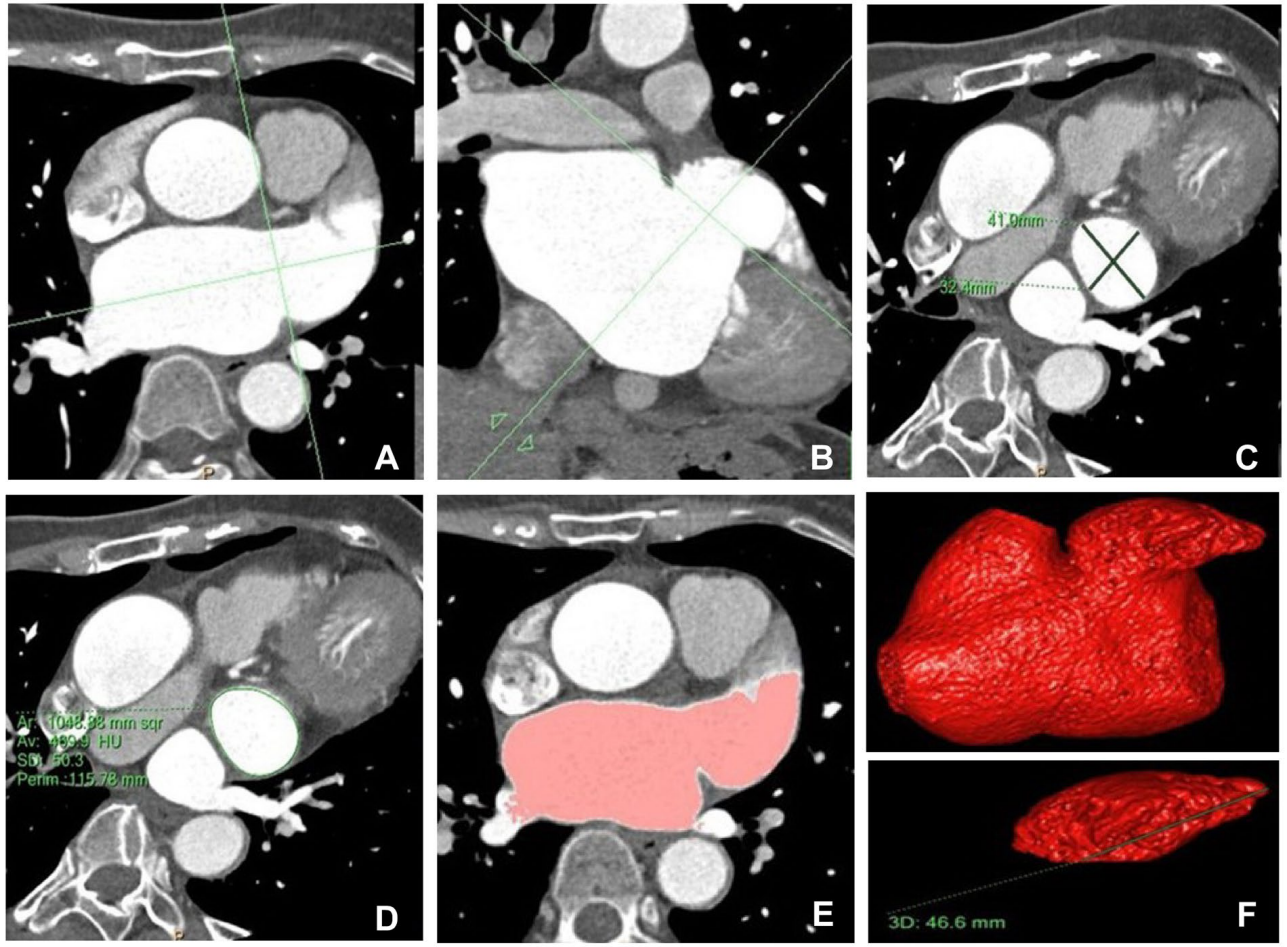
Quantitative measurement of left atrium and left atrial appendage (LAA). (**A–C**) In computed tomographic imaging, the coronary positioning line was put across the conjunction of the left atrium with LAA in the cross section (**A,B**) to obtain the cross section of the orifice of the LAA (C). The major and minor axes of the LAA were measured (**C**). (**D**) The perimeter of the LAA orifice was measured. (**E,F**) The left atrium and LAA imaging was obtained using cardiac function software for measuring the volume of the left atrium and LAA. The LAA depth was measured (F, lower part) from the most distal tip of LAA to the center of the cross section of the LAA orifice. The upper part in F is the left atrium and LAA.

Philips EBW4.5 workstation was used to reconstruct the original image at 5%-95%, with an interval of 10%. The thickness of the reconstructed layer was 0.9 mm and the interval was 0.45 mm. Three-dimensional images of the left atrium and LAA were obtained on the axial images with the post-processing software of cardiac function. The perimeter, major and minor axes of the orifice of LAA were measured putting the coronary positioning line across the conjunction of the left atrium with LAA to obtain the cross section of the orifice of the LAA (Fig. 1). The perimeter and axes of the LAA were measured at the 45% time phase of the cardiac cycle corresponding to the cardiac systolic cycle. In the CT image, the coronary positioning line was put across the conjunction of the left atrium with LAA in the cross section (Fig. 1A&B) to obtain the cross section of the orifice of the LAA (C) for measurement of the major and minor axes of the LAA (Fig. 1C) and the perimeter of the LAA orifice (Fig. 1D). The positioning line was perpendicular to the intersection of the LAA with the left atrium. After multiplane reconstruction and on the coronal location, the positioning line was perpendicular to the conjunction between the LAA and left atrium. The cross-section image of the bottom of the LAA was obtained. The length, short diameter and perimeter of the bottom of the LAA were measured (Fig. 1). The total volume of the left atrium and LAA were automatically calculated. The LAA depth was measured (Fig. 1F, lower part) from the most distal tip of LAA to the center of the cross section of the LAA orifice. The 45% time phase of the cardiac cycle is systolic, and at this period, the LAA has the greatest volume. At 65–85% of the cardiac cycle, the structural parameters of the LAA have minimal changes corresponding to the natural status. The maximal structural parameters of LAA may change with AF. The depth, opening area, diameter lines, and the end diastolic volume of the LAA (i.e. the maximal diameter line of the LAA structure) are significantly greater in patients with than without chronic AF^9^. Therefore, the choice of the 45% of the cardiac cycle can better reflect the structural changes of LAA. The left atrial volume and LAA volume were measured at every phase, and then the maximal and minimal left atrial volume (LAVmax and LAVmin, respectively), maximal and minimal volume of the LAA (LAAVmax and LAAVmin, respectively) were obtained. The LAA ejection fraction and volume (LAAEF and LAAEV, respectively), left atrial ejection fraction and volume (LAEF and LAEV, respectively), left atrial volume strain (LA-VS) and LAA volume strain (LAA – VS) were calculated as follows: LAAEV = LAAVmax-LAAVmin, LAAEF = (LAAVmax – LAAVmin)/LAAVmax × 100%, LAA – VS = (LAAVmax-LAAVmin)/LAAVmin × 100%, LAEV = LAVmax-LAVmin, LAEF = (LAVmax – LAVmin)/LAVmax × 100%, and LA – VS = (LAVmax – LAVmin)/LAVmin × 100%.

### Radiofrequency catheter ablation

Radiofrequency catheter ablation was performed according to the unified standard for circumferential pulmonary vein isolation. The coronal sinus electrodes were inserted into the subclavian vein or femoral vein of the patient, and atrial septal puncture was performed to reconstruct the left atrium-pulmonary vein. The pulmonary veins were isolated and ablated. Two-way conduction block between the pulmonary vein and left atrium was confirmed by electrophysiological stimulation after the operation.

### Postoperative management and follow-up

Surface ECG or dynamic electrocardiogram (DCG) results were obtained at 3, 6 and 12 months after operation. The DCG was 24-hour dynamic ECG to record the condition of the heart within 24 hours including normal and abnormal activity and status. If palpitation symptoms occurred, ECG examination was performed immediately. The endpoint at follow-up was recurrence of AF which was defined as rapid atrial arrhythmia (atrial tachycardia, atrial flutter, atrial fibrillation) recorded by ECG after 3 months of blank period.

### Statistical analysis

SPSS 21.0 statistical software (IBM, Chicago, IL, USA) was used for data analysis. Counting data were expressed as an amount (%) and analyzed with the chi-square test. Mean comparisons between two groups with normal distribution were expressed as mean ± SD (standard deviation) and analyzed by independent sample t-test, and data with non-normal distribution were expressed by Wilcoxon rank-sum test and median (quartile spacing). Univariate and multivariate Cox proportional risk regression models were used to evaluate the risk factors associated with recurrence of AF. Test for collinearity in the variables was included in the model. Strongly correlated variables were excluded. The variance inflation factors were calculated. Independent predictors of recurrence after radiofrequency ablation of AF were analyzed, and adjusted risk ratio (HR) was calculated. The best critical value of predictors of recurrence after AF surgery was calculated by ROC (receiver operating curve) analysis. The statistical significance was set at P < 0.05.

## Results

Eighty-three patients with AF who underwent radiofrequency ablation for the first time were enrolled, including 49 male and 34 female patients with a mean age of 60.36 ± 10.11 and a mean BMI of 25.98 ± 4.06 kg/m^2^. The median follow-up time was 19 (range 4–24) months, and 27 patients (32.5%) relapsed. The patients were divided into two groups: recurrence group (n = 27, mean age 63.26 ± 9.30 year) and the non-recurrence group (n = 56, mean age 58.96 ± 10.26 year).

The clinical data of patients after radiofrequency ablation of AF were compared (Table 1). No significant (P > 0.05) differences existed in the composition of age, sex, BMI, diabetes mellitus, hypertension, coronary heart disease, stroke, transient ischemic attack and duration of AF between the two groups. However, the CHA2DS2-VASc score and heart failure were significantly (P < 0.05) greater in the recurrence than non-recurrence group.

**Table 1:** Clinical data after radiofrequency ablation of atrial fibrillation.

	Recurrence (n = 27)	Non-recurrence (n = 56)	*P*
Age (y)	63.26 ± 9.30	58.96 ± 10.26	0.069
Sex (males)	15(55.6%)	34(60.7%)	0.654
BMI (kg/m^2^)	26.12 ± 3.63	25.90 ± 4.28	0.818
Diabetes Mellitus	4(14.8%)	4(7.1%)	0.476
Hypertension	15(55.6%)	26(46.4%)	0.436
Coronary heart disease	14(51.9%)	23(41.1%)	0.355
Stroke/TIA	2(7.4%)	9(16.1%)	0.456
Heart failure	11(40.7%)	7(12.5%)	0.003
LVEF	61.86(60.80,62.50)	61.47(60.78,62.50)	0.818
CHA_2_DS_2_-VASc	3.00(3.00,4.00)	2.00(1.00, 4.00)	0.013
AF duration (months)	24.00(8, 60)	24.00(6.25,36.00)	0.398

Note: AF, atrial fibrillation; BMI, body mass index; TIA, transient ischemic attack; LVEF, left ventricular ejection fraction.

Quantitative measurement of the morphology and functional parameters of the left atrium and LAA was performed. The LAAVmax, LAAVmin, LAVmax, and LAVmin were significantly greater in the recurrence than non-recurrence group (P < 0.05), and the perimeter, major and minor axes of LAA orifice and LAA depth were also significantly greater in the recurrence than non-recurrence group (Table 2). The LAAEF, LAEF and LAA-VS were significantly (P < 0.05) lower in the recurrence than non-recurrence group (P < 0.05) (Table 2). However, no significant (P > 0.05) differences existed in the LAAEV, LAEV, and LA-VS between the two groups. LAEF, LAAEF, and LAA-VS were all calculated from LAAVmax and LAAVmin. The opening and depth of LAA were strongly related to LAAVmax and LAAVmin. Therefore, only LAEF and LAAEF were retained after removing LAVmax, LAVmin, LAAVmax and LAAVmin. Although LAV correlated with LAVmax and LAVmin and LAAV correlated with LAAVmax and LAAVmin, LAV and LAAV represented LA and LAA morphology, respectively. Therefore, LAV and LAAV were also included in the multivariate analysis. Combined with the P value in Table 1, the measurement contents with P value < 0.2 of the group difference were included in the model, namely: age, CHA2DS2-VASC, and heart failure.

**Table 2:** Parameters of left atrium and LAA in two groups after radiofrequency ablation.

Variables	Non-recurrence (n = 56)	Recurrence (n = 27)	*P*
LAAVmax	8.32 ± 3.64	11.20 ± 4.81	0.011
LAAVmin	3.46(2.38,5.70)	7.02 ± 3.09	0.000
LAAV	15.80(9.60,18.80)	8.53 ± 3.16	0.000
LAAEV	3.80(2.43,5.60)	4.18 ± 1.94	1.000
LAAEF	51.14 ± 12.43	37.21 ± 7.03	0.000
LAA-VS	1.00 (0.71,1.62)	0.61 ± 0.17	0.000
**LAA**			
Orifice perimeter (mm)	81.11 ± 62.87	80.70 ± 17.66	0.013
Orifice major axis(mm)	25.50(21.70,28.90)	28.97 ± 4.91	0.003
Orifice minor axis(mm)	18.11 ± 6.01	22.16 ± 4.34	0.002
Depth(mm)	39.10(32.68,44.15)	47.01 ± 10.33	0.005
LAVmax	114.48 ± 29.98	143.74 ± 36.70	0.001
LAVmin	76.13 ± 32.59	110.24 ± 41.54	0.001
LAV	141.91 ± 34.25	115.65 ± 30.22	0.001
LAEV	38.35 ± 15.36	33.50 ± 12.06	0.218
LAEF	39.61(20.00,48.76)	25.56 ± 12.81	0.013
LA-VS	0.62 ± 0.35	0.28(0.17,0.58)	0.130

Note: LAA, left atrial appendage; LAAVmax, maximal volume of the LAA; LAAVmin, minimal volume of the LAA; LAAEV, LAA ejection volume; LAAEF, LAA ejection fraction; LAA-VS, LAA volume strain; LAVmax, maximal left atrial volume; LAVmin, minimal left atrial volume; LAEV, left atrial ejection volume; LAEF, left atrial ejection fraction; LA-VS, left atrial volume strain.

Univariate Cox proportional risk regression analysis showed that the P value of age was 0.088, so age was not included in the multivariate analysis. LAEF and LAAEF were significant (P < 0.05) risk factors for AF recurrence after radiofrequency ablation (Table 3). The covariance expansion factors of LAAEF and LAEF were calculated, which were 2.069 and 2.018, respectively. Because these factors were less than 5, the LAAEF and LAEF did not have collinearity. CHA2DS2-VASC, LAV, and LAAV were significant (P < 0.05) risk factors for AF recurrence after radiofrequency ablation.

**Table 3:** Univariate hazard model analysis of variables with AF recurrence.

	HR (95% CI)	*P*
Age	1.037 (0.995–1.081)	0.088
CHA_2_DS_2_-VASC score	1.274 (1.025–1.583)	0.029
Heart failure	0.304 (0.140–0.660)	0.003
LAV	1.019 (1.007–1.031)	0.002
LAEF	0.963 (0.932–0.995)	0.026
LAAEF	0.912 (0.868–0.958)	0.000

Note: AF, atrial fibrillation; HR, hazardous ratio; CI, confidence interval; LAEF, left atrial ejection fraction; LAAEF, left atrial appendage ejection fraction.

**Figure 2:**
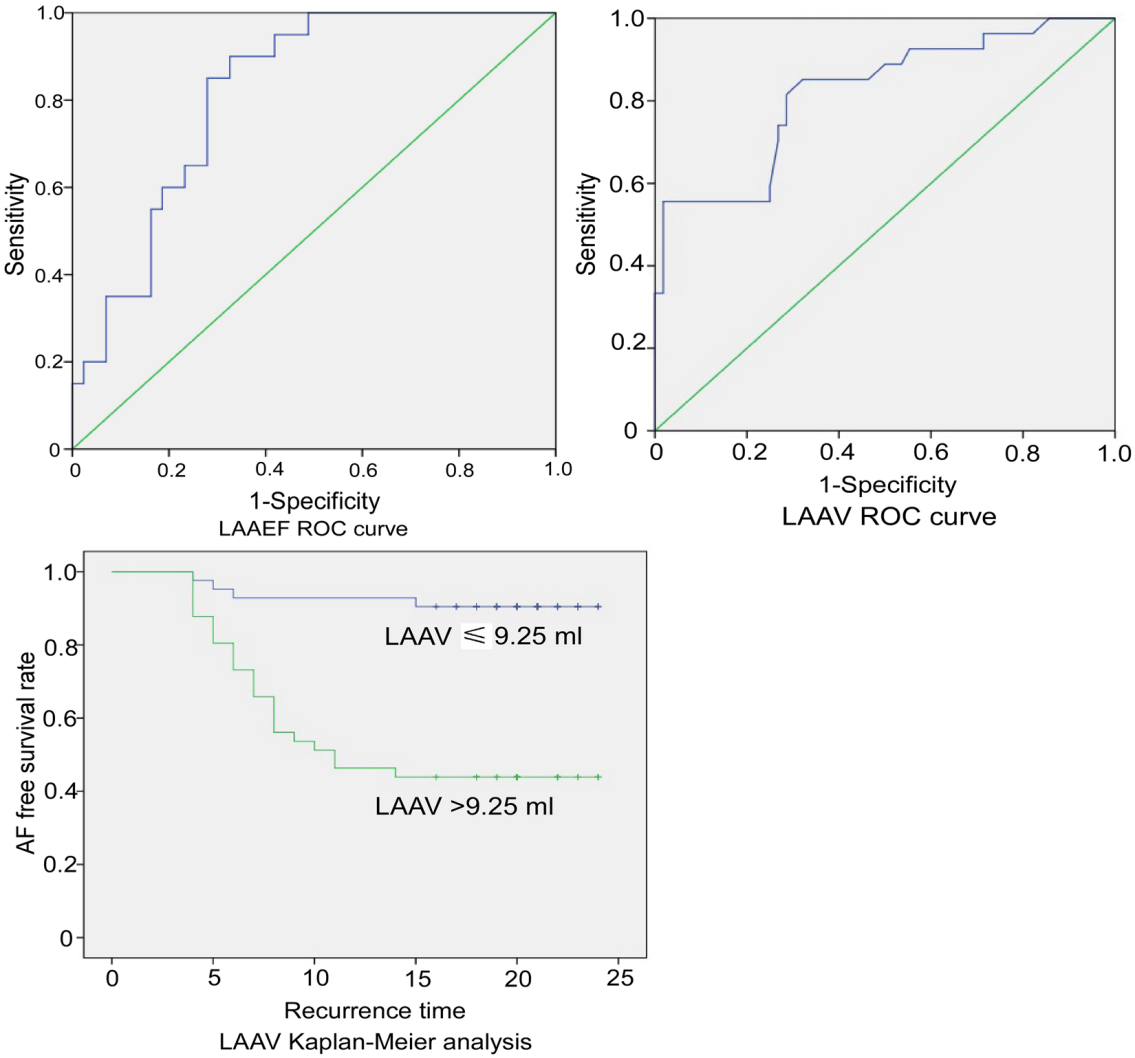
ROC curve analysis of the volume and ejection fraction of the left atrial appendage (LAAV and LAAEF, respectively) and Kaplan-Meier analysis of LAAV. The star indicates the LAAEF or the LAAV with the highest predictive value of recurrence after radiofrequency ablation. The numbers at risk are demonstrated in the Kaplan-Meier analysis of LAAV.

Multivariate analysis revealed LAAEF (HR: 0.79, 95% CI: 0.66–0.95, P = 0.01) and LAAV (HR: 1.160, 95% CI: 1.095–1.229, P < 0.001) to be significant independent predictors of recurrence after radiofrequency ablation of AF (Table 4). For every 1% increase in LAAEF, the AF recurrence rate after radiofrequency ablation is decreased 21%, whereas for every 1 ml increase in the LAA volume, the risk for AF recurrence after radiofrequency ablation increased 1.16 times. ROC curve analysis showed that LAAEF < 44.68% had the highest predictive value for recurrence after radiofrequency ablation (area under curve 0.817), with the sensitivity of 90% and specificity of 67.4%, whereas LAA volume >9.25 ml had the highest predictive value for AF recurrence after radiofrequency ablation (area under curve 0.82), with the sensitivity of 85.2% and specificity of 67.9% (Fig. 2). Among all 27 patients with AF recurrence, only four patients had the LAA volume ≤9.25 ml. Kaplan-Meier curve analysis (Fig. 2) further showed that patients with LAA volume >0.25 ml had a greater recurrence rate after radiofrequency ablation (P < 0.001).

**Table 4:** Multivariate hazard model analysis of variables with AF recurrence.

	HR (95% CI)	*P*
CHA2DS2-VASC score	—	0.225
Heart failure	—	0.077
LAV	—	0.698
LAEF	1.117(0.942–1.325)	0.204
LAAEF	0.790(0.657–0.950)	0.012
LAAV	1.160(1.095–1.229)	0.000

Note: AF, atrial fibrillation; HR, hazardous ratio; CI, confidence interval; LAEF, left atrial ejection fraction; LAAEF, left atrial appendage ejection fraction.

## Discussion

Catheter radiofrequency ablation is an effective therapeutic approach for the treatment of AF, especially for refractory patients who have episodes of arrhythmia despite antiarrhythmic medicine therapy. For patients with paroxysmal AF, catheter radiofrequency ablation can be used as a first-line therapy. However, recurrence of AF remains an important issue. Exploring the risk predictors of AF recurrence is critical to identifying high-risk patients following radiofrequency ablation, thus providing a basis for preoperative selection of treatment methods and improving the ablation success rate. The recurrence rate following AF radiofrequency ablation in this study was 32.5%, which was consistent with that reported in previous research^10^. Our study showed that the LAVmax, LAVmin, LAAVmax, and LAAVmin were all significantly elevated in patients with recurrence compared with those without recurrence, whereas the LAAEF and LAEF significantly decreased in patients with recurrence compared with those of non-recurrence, with the LAAEF and LAAV being two significant independent factors to predict recurrence following catheter radiofrequency ablation. Previous studies have also demonstrated that increased volumes of left atrium and LAA but decreased ejection fraction of the left atrium and LAA are risk predictors of recurrence after radiofrequency ablation of AF^5,6^.

Left atrial overload may result in structural remodeling, atrial hypertrophy and stretch, and subsequent ionic current alterations and electrical remodeling^11^. Cardiac endothelin-1 expression correlates with enlarged left atrium size and responds to the wall stress caused by enlarged left atrium, thus promoting hypertrophy of myocytes and fibrosis of myocardial interstitium. Atrial fibrosis in patches can cause slow conduction and change dynamic repolarization in some areas, consequently shifting the initiation focus and AF maintenance to the left atrium from the pulmonary veins^12,13^. In this case, radiofrequency ablation may be insufficient, resulting in an increased rate of recurrence. Left atrial enlargement is able to independently predict a new AF onset^14^, and the left atrium diameter has been reported as a predicting factor for AF recurrence following radiofrequency ablation^15,16^. However, the anterior-posterior diameter of the left atrium is not regarded to reflect the real size of left atrium. Abecasis *et al*.^17^ demonstrated that the left atrium volume rather than the anterior-posterior diameter of left atrium was related to AF recurrence following radiofrequency ablation. In a study investigating the relationship of left atrium volume with AF recurrence following ablation^18^, Helms *et al*. proved that the volume of the left atrium was significantly increased in recurrence and that only the volume of the left atrium and number of cardiovascular diseases were significantly associated with more recurrences, with patients having left atrial volumes > 135 ml being very likely to experience AF recurrence after radiofrequency ablation. In a meta-analysis^11^, Nioku *et al*. proved that the volume rather than the diameter of left atrium is a more accurate parameter in predicting AF recurrence after radiofrequency ablation.

The LAA is a frequent location of thrombosis in AF patients, and significantly larger LAA and left atrium are present in AF patients than those without AF. Expanded LAA may cause poor contraction and elevated risk of thrombosis of both the LAA and the whole system^19^. Successful radiofrequency ablation to eliminate AF will significantly decrease the orifice, neck and length of the LAA and reverse the LAA neck shape which is less eccentric in AF patients^19^. Nonetheless, AF recurrence following ablation will lead to progressive dilation of the LAA but no change in the eccentricity of the LAA orifice and neck. The LAA has a blind pouch and a relatively narrow orifice to the main chamber of the left atrium. When the LAA is remodeled in AF, the LAA is stretched and loses its compliance and contractility, and the shape of the LAA neck becomes rounder. Moreover, less cardiomyolysis and less interstitial fibrosis are presented in LAA than in the left atrial main chamber in AF. After radiofrequency ablation, the size of LAA may decrease faster than that of the left atrium^20^. AF recurrence following catheter ablation will further dilate both the left atrium and LAA because prolonged AF will cause cardiomyocyte hyperplasia, death and fibrosis, resulting in enlargement of cardiac chambers and tachyarrhythmia attack.

For the LAA volume, He *et al*. demonstrated in a study with transesophageal echocardiography for measuring the LAA volume and function that the LAAVmax and LAAVmin were markedly increased in recurrence while the function parameters including LAAEF, LAA flow emptying velocity and summit filling velocity were significantly decreased, with the multivariate analysis showing the left atrial volume index, LAAEF and LAA flow emptying velocity to be significant factors to predict paroxysmal AF recurrence following radiofrequency ablation^21^. In a study with multi-detector CT scanning investigating the LAA volume in association with AF recurrence following radiofrequency ablation^22^, Shiozawa *et al*. found that both greater LAA volumes and orifice areas were exhibited in recurrent AF patients while the diameter and volume of the left atrium did not have significant differences between patients with and without recurrence. All patients possessing a LAA volume greater than 25 ml experienced AF recurrence. As an embryonic remnant of the left atrium, the LAA plays a very important role in adjusting heart rate and fluid balance via an expandable reservoir function, a capacity to secret atrial natriuretic peptides, and sensitive receptors of stretch^22–24^. The LAA is distensible at a greater degree than the left atrium, and the LAA volume is a dependable parameter to determine the left atrial conditions in both function and structure in early AF. After analyzing the relationship between LAA volume and AF recurrence following catheter ablation^25^, Pinto *et al*. pointed out that the volume of the LAA on cardiac CT may be able to independently predict recurrence of paroxysmal and persistent fibrillation after first ablation and that a volume of the LAA more than 8.825 ml may accurately predict recurrent AF.

The left atrium may act as a contractable pump providing up to a third of the volume of the left ventricle, as a reservoir collecting pulmonary venous blood in the right ventricular systolic period, and as a channel to pass stored blood from the left atrium to the left ventricle^26^. The LAEF reflects left atrial conduit and contracting function while the LAAEF indicates the contracting function only. In our study, the LAAEF and LAEF significantly decreased in patients with recurrence compared with those without recurrence. Impaired emptying fraction of the left atrium (≤20%), decreased emptying velocity (≤20 cm/sec) and fraction (≤20%) of the LAA were significantly correlated with recurrent AF after ablation^6^. A low LAA peak flow velocity and upward velocity of the LAA apex wall before surgery have been reported to relate to recurrent AF following the initial ablation for persistent AF^27,28^. Chin *et al*.^29^ have reported that the LAEF and LAAEF have significantly decreased in patients with AF recurrence after one year, indicating that AF recurrence may worsen left atrial function. After studying the risk-predicting factors for AF recurrence following revised endoscopic ablation at two years of follow-up, An *et al*.^30^ found that the duration of AF, the diameter of left atrium, and LAAEF could significantly independently predict AF recurrence. Decreased LAEF and LAAEF in patients with AF recurrence indicate decreased function in the reservoir and contraction of the left atrium and LAA, which may denote enlarged volume of the left atrium and LAA, and consequently the anatomical parameters of the LAA also increase including the perimeter, major and minor axes of LAA orifice and LAA depth. These parameters were significantly greater in patients with recurrence compared with those without recurrence in our study. Age is also an important factor for AF recurrence^31^. The older the patient, the more likely the presence of AF recurrence after radiofrequency ablation. In our study, patients with AF recurrence were not significantly different in age from those without recurrence, and that is why multivariable analysis did not show age as a significant factor for recurrence.

AF originating from the LAA is one important index for AF recurrence following radiofrequency ablation. It has been reported that 27% of patients with recurrent arrhythmias after radiofrequency ablation of AF were associated with LAA^4^. Further investigation has found that LAA isolation can reduce the recurrence rate of arrhythmia^4^. Therefore, the relationship between LAA function and AF recurrence after radiofrequency ablation is very important. In our study, the LAAEF rather than the LAA volume is a significant factor to predict AF recurrence following radiofrequency ablation, suggesting that LAA function may be more important than LAA volume in predicting recurrence after radiofrequency ablation of AF. Ejection fraction is a better parameter in revealing the rate of volume decrease after ejection of blood and indicating the contractile function of LAA.

AF causes the structure of LAA remodeling like fibrosis, which is related to AF relapse after radiofrequency ablation. The LAA has abundant comb muscles, which have stronger autonomic contraction function than muscles in the left atrium. Changes in the pressure and volume of the left atrium can help regulating its systolic and diastolic function^32^. In AF patients, the left atrium loses its rhythm, and the ejection function is decreased. To alleviate increased intra-atrial pressure, the myocardia of LAA are structurally remodeled. The degree of LAA remodeling in recurrent patients of AF following radiofrequency ablation is greater than in those without recurrence^33^. LAA remodeling may lead to electrophysiological changes in LAA. It has been shown that left atrial fibrosis is associated with conduction abnormalities and aggravates recurrence of AF^34^. Our research showed that, compared to the left atrium, fibrosis of LAA myocardia represented by LAA-VS was more important in recurrence, with significantly greater LAA-VS in the recurrence than non-recurrence group. Displaying myocardial fibrosis of left atrium and LAA is difficult because the fibrosis is too delicate to be measured. Strain imaging of the magnetic resonance imaging can be used to assess fibrosis of myocardia, however, it needs special software, which limits its clinical usage. In our study, the volume strain of left atrium and LAA were calculated with use of the 256-slice CT, which can be easily performed in regular cardiac CT scanning.

Transesophageal echocardiography (TEE) stands for the gold standard in measuring LAA parameters. With high spatial resolution and high repeatability, multi-slice CT (MSCT) is well correlated with TEE in the measurement of the LAA volume and function^35^. It has been shown that LAAEF measured by TEE can predict the sinus rhythm status in AF patients post treatment^21^. After examining paroxysmal AF in 80 patients by TEE prior to radiofrequency ablation, He *et al*.^21^ found that high LAAEF was a protective factor for preventing AF recurrence. The combined model of left atrial volume index, LAEF and LAA-emptying velocity (LAVI + LAAEF + LAAeV) was effective in predicting paroxysmal AF recurrence^21^. It has been shown that LAAEF < 30% measured by TEE is an independent index for predicting non-valvular AF recurrence following cardioversion^36^. However, the relationship between LAA function measured by multi-slice spiral CT and recurrence after radiofrequency ablation has rarely been reported. In our study, the 256-slice spiral CT was used to measure LAA function for the first time to predict AF recurrence after radiofrequency ablation, and our results showed that LAAEF was also an independent factor to predict AF recurrence following radiofrequency ablation, with LAAEF < 44.68% having the highest value for predicting AF recurrence following radiofrequency ablation.

The CHADS2 and the CHA2DS2-VASc systems are affirmative risk stratification models for predicting vascular events and ischemic strokes in non-valvular AF^8,37^. There are some values in these scores for predicting left atrial ablation outcomes. Larger left atrial volume, diabetes, dyslipidemia, coronary artery disease, class III antiarrhythmic drugs, CHADS2 and CHA2DS2-VASc scores had univariately associated with AF recurrence after left atrial ablation, and both CHADS2 and CHA2DS2-VASc could independently predict AF recurrence^8^. The CHA2DS2-VASc scores, combined with other clinical parameters of heart failure, advanced age, hypertension, and diabetes, are dependable parameters for predicting AF recurrence after left atrial ablation. In our study, however, the CHA2DS2-VASc score system did not perform better than LAAEF and increased LAA volume in predicting AF recurrence following catheter ablation even though this score was significantly increased in AF recurrence compared with non-recurrence after catheter ablation.

Addition of LAA ligation using the percutaneous endoepicardial closure system to common radiofrequency ablation can improve the ablation success rate but decrease the recurrence rate^38–40^. The AMEZE trial may be a good approach to determine if LAA ligation as an adjunctive treatment approach to pulmonary venous isolation is able to increase the efficacy of sinus rhythm maintenance in persistent AF or long-term persistent AF^39^. In addition, LAA electrical isolation combined with standard ablation helps achieving freedom from various atrial arrhythmias in persistent AF or long-term persistent AF^40^. In the study of LAA ligation combined with ablation for persistent AF by Lakkireddy *et al*.^38^ enrolling a total of 138 patients with 69 patients in the group with additional LAA ligation (LARIAT), the primary outcome without AF at 1 year off antiarrhythmic treatment following one ablation procedure was better in the LARIAT group (65% vs. 39%, P < 0.01) than in the group applying radiofrequency ablation alone, with significantly more patients in the group using ablation only experiencing repeated ablation due to AF recurrence (33% vs. 16%, P = 0.018). Radiofrequency ablation combined with LAA ligation may exclude the LAA both mechanically and electrically and help improving the prognoses of radiofrequency ablation through eliminating the triggers and changing the substrate of the LAA.

Some limitations may exist in our study including single center study, only Chinese ethnicity enrolled, a small cohort of patients, retrospective nature and non-randomization. Future research will need to overcome these limitations for better outcomes.

In summary, the left atrial volume, the volume and morphology of the left atrial appendage have all significantly increased, whereas the ejection fraction and volume strain of both the left atrium and atrial appendage have both significantly decreased in recurrence than in non-recurrence after radiofrequency ablation. The ejection fraction and volume of the left atrial appendage are significant independent factors to predict recurrence of atrial fibrillation following radiofrequency ablation.
